# Born into adversity: psychological distress in two birth cohorts of second-generation Irish children growing up in Britain

**DOI:** 10.1093/pubmed/fdt034

**Published:** 2013-04-17

**Authors:** J. Das-Munshi, C. Clark, M.E. Dewey, G. Leavey, S.A. Stansfeld, M.J. Prince

**Affiliations:** 1 Section of Epidemiology, Department of Health Services Research and Health of Populations, Institute of Psychiatry, King's College London, London SE5 8AF, UK; 2 Centre for Psychiatry, Queen Mary University of London, Barts and the London School of Medicine, London EC1M 6BQ, UK; 3 Bamford Centre for Mental Health, University of Ulster, Londonderry, UK

**Keywords:** BCS70, Irish, migration, minority ethnic mental health, NCDS

## Abstract

**Background:**

Worldwide, the Irish diaspora experience health inequalities persisting across generations. The present study sought to establish the prevalence of psychological morbidity in the children of migrant parents from Ireland, and reasons for differences.

**Methods:**

Data from two British birth cohorts were used for analysis. Each surveyed 17 000 babies born in one week in 1958 and 1970 and followed up through childhood. Validated scales assessed psychological health.

**Results:**

Relative to the rest of the cohort, second-generation Irish children grew up in material hardship and showed greater psychological problems at ages 7, 11 (1958 cohort) and 16 (both cohorts). Adjusting for material adversity and maternal psychological distress markedly reduced differences. Relative to non-Irish parents, Irish-born parents were more likely to report chronic health problems (odds ratio [OR]: 1.29; 95% confidence interval [CI]: 1.08–1.54), and Irish-born mothers were more likely to be psychologically distressed (OR: 1.44; 95% CI: 1.13–1.84, when child was 10). Effect sizes diminished once material adversity was taken into account.

**Conclusions:**

Second-generation Irish children experienced high levels of psychological morbidity, but this was accounted for through adverse material circumstances in childhood and psychological distress in parents. Public health initiatives focusing on settlement experiences may reduce health inequalities in migrant children.

## Introduction

Research accounting for the intergenerational ‘transmission’ of health inequalities to second-generation ethnic minority groups remains scant, especially, as regards to the use of prospective data from birth cohorts. In particular, the role of migration and settlement in accounting for the transmission of health disadvantages from parent to child has been little described. Previous studies have reported differences in mental health among ethnic minority children and have however failed to assess potential aetiological mechanisms.^1^ Examining these mechanisms using a life-course informed perspective may help in understanding the social risks related to the environment, which migrant parents move into, and into which their children are raised. This is important as social and health disadvantages in childhood may continue to influence downstream adult health many years later^2–4^, and offers the possibility for early intervention.

Often excluded from discussions of ethnic minority health disparities, people of Irish descent constitute one of the largest ethnic minority groups living in Britain.^5^ Of note, Irish people living in Britain experience elevated mortality and poorer self-rated health and limiting long-term illness, which has persisted into second and third generations, despite improvements in socioeconomic circumstances.^6–8^ Previous research on Irish-born and second-generation Irish people living in Britain has suggested that the prevalence of depression^9,10^ and suicidality^11^ are elevated compared with rates in Ireland^12^ and compared with non-migrant groups living in Britain.^7^

Although controversial, it has been suggested that the poorer health of Irish-born migrants in Britain may be due to negative selection effects (‘unhealthy migrant selection effect’), with Ireland's geographical proximity to Britain and shared language presenting fewer barriers to migration.^13,14^ For example, a working paper by Delaney *et al.*,^13^ using data from England and Ireland, noted that Irish-born migrants to England, born in the periods spanning 1920–60, were of a shorter stature, lower educational attainment as well as were also more likely to report poorer self-rated health and have poorer mental health than age-matched Irish-born counterparts remaining in Ireland, and age-matched English-born counterparts living in England. As education and height may be taken as proxy indicators of pre-migration and/or childhood experiences, the authors of this report suggest that Irish migration at this time was driven by large differences in income between England and Ireland, such that people who were of a lower educational attainment and who, therefore, were more likely to have experienced childhood disadvantage themselves were more likely to have emigrated from the poorer regions of Ireland for work.^13^ The author's analysis of health of Irish-born people living in England suggested that health inequalities persisted after adjustment for factors related to the post-migration settlement context.^13^ The authors note that this demographic picture shifted from the late 1970s when differences in mean income between the two countries had lessened, favouring more skilled Irish-born migrants with higher levels of education, representative of wider regions of Ireland, to migrate.^13^

In addition, migration may be perceived as relatively straightforward, simple and/or temporary by Irish-born migrants^10,15^; poorly planned migration may be associated with adverse health consequences, such as depression, particularly, if post-migration factors such as social support are lacking.^15^ The health risks of informal migration in Irish-born migrants have been compared with other settings in Europe, where minimal legal restrictions to movement and low-cost travel predisposes to unplanned migration. This is associated with adverse health consequences.^10,16^

Even if selection effects at least partially accounted for the poorer health of Irish-born migrants to Britain, this would not explain the stark health inequalities, which have been consistently reported in second-generation Irish people; one would expect health inequalities to approximate to that of the receiving country, across generations. To date, there have been very few prospective studies which have examined the childhood experiences of second-generation ethnic minority children and experiences of settlement, which may account for potential health inequalities.

Thus, we sought to establish the prevalence of psychological morbidity in second-generation Irish children born in Britain, using two prospective birth cohorts, the National Childhood Development Survey (NCDS), and the 1970 Birth Cohort (BCS70). We sought to assess whether any differences in psychological health might be accounted for either through adversity related to settlement, or through poorer parental health. We assessed parental health through contemporaneous measures, it was not possible in this dataset to assess the pre-migration health of Irish-born parents.

We tested the following hypotheses:

- Children of Irish-born migrants are more likely to have a higher prevalence of psychological distress, relative to that of non-migrant parents.
- An excess in psychological morbidity in children of Irish-born parents compared with non-Irish parents is explained by parental social and health inequalities.

## Methods

### Sample

The cohorts comprised 17 000 children born in England, Scotland or Wales, over 1 week in 1958 (NCDS) and 1970 (BCS70), respectively. BCS70 included children born in Northern Ireland. We excluded this group as they were not followed up in the later sweeps of the cohort. We restricted the study samples to include children of parents who were born in Britain, Ireland or Northern Ireland, and excluded children not born in Britain. At ages 11 and 16 of the NCDS, and at birth in the BCS70, parents reported their country of birth. Cohort members with one or both parents reporting that they were born in Ireland or Northern Ireland were classified as second-generation Irish. In NCDS, excluding non-responders, kappa assessing the reliability of parental responses to this question between the two sweeps was 0.97.

### Measures

#### Social inequality

In the NCDS, at ages 7, 11 and 16, and in the BCS70 at age 5, parents reported if they had sole access to household amenities (indoor toilet, bathroom and hot water) or lived in overcrowded housing (1+persons/room). In NCDS (ages 7, 11 and 16), parents were asked to report if they had experienced serious financial hardship in the previous year. In NCDS (age 7), parents were asked if they had faced family housing difficulties within the previous year. At ages 11 and 16, parents were asked if their child received free school meals.

Within the BCS70, at ages 5, 10 and 16, parents reported if they owned a car or van. Parents were also asked if they had damp in their housing, and if their child received free school meals (age 10) or if the family could afford to heat their entire home during the winter (age 16). A social rating of neighbourhoods (‘well to do/affluent’ versus ‘average’/‘poor’/‘rural’) was taken at age 5, judged by interviewers. Parents in the BCS70 were asked about neighbourhood characteristics (age 10) and whether or not they lived in social housing (age 16).

#### Parental health

In the NCDS, parents were asked to report parental chronic health problems when cohort members were aged 11 and 16. In the BCS70, at ages 5, 10 and 16, mothers answered questions from the self-rated 24-item Rutter Malaise inventory, which assesses mental health.^17^ The scale has adequate reliability, with comparable results across gender and socioeconomic position.^18^ At age 5, mothers could answer each item with a ‘yes’ or ‘no’ response (yielding a maximum score of 24). At age 10, a visual analogue scale ranging from 0 to 100 was used. At age 16, a three-point Likert scale (maximum score: 48) was used. To aid comparability across sweeps, summed scores were dichotomized; the top fifth of each distribution was taken as indicating ‘moderate’/‘severe’ psychological disturbances in the parent.

#### Cohort members' psychological health

In the NCDS, when cohort members were 7 and 11 teachers rated their psychological health using the Bristol Social Adjustment Guide^19^; at age 16, the teacher-rated Rutter-B was used.^20^ Both instruments have been well validated.^19–21^ Teachers rated statements regarding the child's behaviour and emotional adjustment by endorsing items on a Likert scale. In the BCS70, when cohort members were 5, 10 and 16, their parents rated emotional or behavioural problems, using the Rutter-A, which have good inter-rater and test–retest reliability.^17^ Questions were rated using a Likert scale, at ages 5 and 16. At age 10, a visual analogue scale was used. Summed scores on each scale resulted in a continuous measure.

### Statistical analysis

Analyses were conducted in STATA-IC 10.1.^22^ In main models, parental migration history was the independent variable with child's psychological symptom count as the dependent variable. Wald tests assessed strengths of association.

#### Data reduction in material hardship through principal components analysis

Principal components analysis (PCA), specifying one component, was used to extract latent constructs for material adversity.^23^ Prior to factor analysis, the Kaiser–Meyer–Olkin measure of sampling adequacy was found to be adequate. Values for the Kaiser–Meyer–Olkin measure of sampling adequacy range from 0 to 1, values closer to 1 are preferred and values of 0.6 are at the lower limit of acceptable. Bartlett's test of sphericity tests the null hypothesis that variables are uncorrelated. Bartlett's test rejected sphericity. In the NCDS, at ages 7, 11 and 16, following PCA, the first component accounted for 42, 40 and 40% of the total variance, respectively. In the BCS70, at ages 5, 10 and 16, the first component accounted for 38, 45 and 43% of the variance, respectively. The weights for each material hardship indicator were used to generate a composite hardship index for each of the sweeps in the two cohorts.^23^

#### Analysis of health and psychological morbidity

Binary outcomes (maternal psychological morbidity and parental chronic illness and death) were modelled using multivariable logistic regression. For the variable ‘total childhood psychological symptom count’, examination of the summed distribution of symptoms indicated over-dispersion, with right skew; therefore, negative binomial regression with and without zero inflation was used for analysis. The underlying assumption of zero-inflated negative binomial regression is that the population comprises two groups, one which is ‘certain zero’ and the other in which members have symptom counts ≥0. The probability that an observed zero is part of the ‘certain zero’ part is modelled with a logistic regression, and the part of the model where counts ≥0 is modelled with a negative binomial regression approach. Derived coefficients were exponentiated, leading to a ‘count ratio’ (CR) with 95% confidence intervals. Migration history and cohort member's gender were entered into the zero-inflation parts of the model. Residuals of the two models (negative binomial regression versus negative binomial regression with zero inflation) were compared using fit statistics, which included the Bayesian Information Criterion, Akaike Information Criterion, log-likelihood and Vuong tests.

## Results

### Demographic information

Six per cent of participants were second-generation Irish in both cohorts. Fifty-two per cent of both cohorts were males. In the NCDS, cohort members were missing information on parental origins due to not being present at the sweeps when this question was asked (ages 11 and 16; *n* = 2038), or being present but not responding (*n* = 1003). Of the 3041 cohort members missing this information, 27% (*n* = 835) had died, and 20% (*n* = 603) had emigrated, at age 16. Reasons for non-response were equivalent between second-generation Irish cohort members and the rest of the sample (Supplementary data, Table S1). Children missing information on parental country of birth were more likely to return incomplete information in general, as these children were missing due to attrition (Supplementary data, Table S2).

In the BCS70, 737 children were missing information on parents' country of birth, of whom the majority (97%) were not present at baseline, when this question was asked (Supplementary data, Table S1). In the BCS70, second-generation Irish cohort members were more likely to be missing information at Sweeps 1–3 due to ‘refusal’ or ‘other’ reasons (Supplementary data, Table S1). In the BCS70, children missing information on parental origins were similar to children not missing this information with respect to gender, but were more likely to return incomplete information for social class at birth, and tenure at age 5 (Supplementary data, Table S3). At age 16, there was a similar tenure profile in children missing information on parental origins and children not missing this information (Supplementary data, Table S3).

Response rates for the NCDS were good, with 83% of living cohort members providing data at age 16. Response rates, at age 16, in the BCS70, were reduced by the national teachers' strike^24^ and was 69% of those children who were still alive.

### Indicators of childhood adversity

Table 1 summarizes that, at each sweep in both cohorts, second-generation Irish children grew up under conditions of marked material adversity compared with British non-migrant children. Mean scores on the composite hardship variable, derived through PCA, were also greater in Irish children (Supplementary data, Table S4).

**Table 1:** Childhood adversity in second-generation Irish children relative to the rest of the cohort; 1958 and 1970 British birth cohorts

	*1958 Birth cohort (*N* = 13 724)*				**x*^2^, *P-*value*		*BCS70 (*N* = 14 854)*				**x*^2^, *P*-value*
	*Country of birth of parent*						*Country of birth of parent*				
	*England, Scotland, Wales*		*Northern Ireland, Ireland*				*England, Scotland, Wales*		*Northern Ireland, Ireland*		
	**n**	*%*	**n**	*%*			**n**	*%*	**n**	*%*	
Age 7						Age 5					
Access to household amenities						Access to household amenities					
Sole use of all^a^	9535	82	502	75	23.42, <0.001	Sole use of all^a^	10 372	95	492	93	6.74, 0.009
None or shared use^a^	2047	18	168	25		None or shared use^a^	495	5	37	7	
Missing	1351		121			Missing	3155	23	303	36	
Overcrowding						Overcrowding					
<1 person/room	6576	59	246	39	91.16, <0.001	<1 person/room	9245	84	384	72	50.34, <0.001
>1 person/room	4646	41	381	61		>1 person/room	1788	16	149	28	
Missing	1711	13	164	21		Missing	2989	21	299	36	
Family financial difficulties						Ownership of van or car					
No	9628	92	491	83	65.74, <0.001	Yes	7978	71	322	59	38.45, <0.001
Yes	822	8	103	17		No	3189	29	223	41	
Missing	2483	19	197	25		Missing	2855	20	287	34	
Family housing difficulties						Social rating of neighbourhood					
No	10 296	93	535	84	70.90 <0.001	Well-to-do/affluent	2585	18	71	9	179.85, <0.001
Yes	763	7	101	16		Rural or average	7588	54	370	44	
Missing	1874	14	155	20		Poor	654	5	84	10	
						Missing	3195	23	307	37	
Age 11						Age 10					
Access to household amenities						Ownership of van or car					
Sole use of all^a^	10 265	88	594	85	4.17 0.041	Yes	8415	76	286	57	97.03, <0.001
None or shared use^a^	1391	12	101	15		No	2642	24	218	43	
Missing	1277	10	96	12		Missing	2965	21	328	39	
Overcrowding						Neighbourhood characteristics					
<1 person/room	7256	62	318	46	70.50 <0.001	Village/rural	7134	65	216	43	100.42, <0.001
>1 person/room	4494	38	377	54		Council/Urban/Other	3925	35	291	57	
Missing	1183	9	96	12		Missing	2963	21	325	39	
Serious financial hardship in the last year						Damp in housing					
No	10 196	89	553	84	20.57, <0.001	No	9179	82	402	78	6.85, 0.009
Yes	1231	11	109	16		Yes	1978	18	115	22	
Missing	1506	12	129	16		Missing	2865	20	315	38	
Free school meals						Free school meals					
No	10 284	90	564	83	34.83 <0.001	No	9463	85	383	75	34.94, <0.001
Yes	1113	10	114	17		Yes	1678	15	126	25	
Missing	1536	12	113	14		Missing	2881	21	323	39	
Age 16						Age 16					
Access to household amenities						Ownership of van or car					
Sole use of all^a^	9179	94	527	94	0.00, 0.996	No	1171	16	61	24	11.83, 0.001
None or shared use^a^	609	6	35	6		Yes	6060	84	189	76	
Missing	3145	24	229	29		Missing	6791	48	582	70	
Overcrowding						Resident in council flat					
<1 person/room	6813	70	294	52	77.12, <0.001	No	3675	84	120	77	5.48, 0.019
>1 person/room	2981	30	272	48		Yes	702	16	36	23	
Missing	3139	24	225	28		Missing	9645	69	676	81	
Serious financial hardship over the last year						Damp in house					
No	8729	90	461	82	34.35, <0.001	No	6564	90	229	90	0.08, 0.777
Yes	949	10	98	18		Yes	702	10	26	10	
Missing	3255	25	232	29		Missing	6756	48	577	69	
Free school meals						Proportion of home family can afford to heat in winter					
No	8869	91	469	83	40.13 <0.001	Proportion of house	5288	67	167	59	9.21, 0.002
Yes	911	9	99	17		Whole house	2556	33	117	41	
Missing	3153	24	223	28		Missing	6178	44	548	66	

^a^Household amenities were: indoor bathroom, indoor toilet and hot water.

### Parental health

In the NCDS, Irish-born parents were more likely to report a chronic health condition, when their child was aged 11 or 16 (Table 2). This difference was reduced, and strengths of associations diminished, when material adversity indicators were taken into account. In the BCS70, there was strong evidence to suggest that Irish-born mothers were more likely to be psychologically distressed when their child was 5 or 10 years with a suggestion for increased risk at age 16 also (Table 2). When indicators for material hardship were taken into account, this excess risk was reduced and the strength of associations diminished.

**Table 2:** Parental health

	*NCDS (1958 British birth cohort) (chronic illness in either parent when child aged 11 or 16)*											
	*Both parents born in England, Scotland, Wales*		*Irish-born parents*		*Model 1*				*Model 2*			
					*Association with chronic illness in Irish-born parents relative to parents in the rest of cohort*				*Association with chronic illness in Irish-born parents relative to parents in the rest of cohort, adjusted for hardship*			
	**n**	*%*	**n**	*%*		*OR*	*95% CI*	**P*-value*		*OR*	*95% CI*	**P*-value*
No chronic illness	9077	79	517	74	Non-Irish parents	1.00	Ref.		Non-Irish	1.00	Ref.	
One or more chronic illness	2359	21	177	26	Irish-born parents	1.35	1.08– 1.70	0.01	Irish-born parents	1.09	0.86 –1.38	0.48
Missing data	1497		97						Hardship, 11^b^	1.33	1.25 –1.42	<0.001 (trend)
									Hardship, 16^b^	1.33	1.25 –1.42	<0.001 (trend)

	BCS70 (1970 British birth cohort) (maternal psychological health, when child aged 5, 10 and 16)											
	Both parents born in England, Scotland, Wales		Irish-born parents		Model 1				Model 2			
					Associations with psychological distress in Irish-born mothers relative to mothers in the rest of cohort				Associations with psychological distress in Irish-born mothers relative to mothers in the rest of cohort, adjusted for hardship			
	*n*	%	*n*	%		OR	95% CI	*P-*value		OR	95% CI	*P*-value
Age 5 (1975)												
Mother not distressed	7241	83	316	77	Non-Irish mother	1.00	Ref.		Non-Irish mother	1.00	Ref.	
Mother psychologically distressed^a^	1508	17	97	23	Irish-born mother	1.43	1.12–1.83	<0.001	Irish-born mother	1.23	0.95–1.58	0.11
Missing data	5273		419						Hardship, 5^b^	1.57	1.49–1.66	<0.001 (trend)
Age 10 (1980)												
Mother not distressed	6646	81	269	75	Non-Irish mother	1.00	Ref.		Non-Irish mother	1.00	Ref.	
Mother psychologically distressed^a^	1556	19	91	25	Irish-born mother	1.43	1.11–1.83	0.01	Irish-born mother	1.13	0.88– 1.47	0.34
Missing data	5820		472						Hardship, 10^b^	1.67	1.59–1.76	<0.001 (trend)
Age 16 (1986)												
Mother not distressed	4780	83	153	80	Non-Irish mother	1.00	Ref.		Non-Irish mother	1.00	Ref.	
Mother psychologically distressed^a^	975	17	38	20	Irish-born mother	1.53	0.86–2.74	0.15	Irish-born mother	1.46	0.80–2.64	0.21
Missing data	8267		641						Hardship, 16^b^	1.50	1.37–1.64	<0.001 (trend)

^a^Rutter Malaise inventory scores in top 20th centile.

^b^Per SD increase in composite hardship indicator.

### Mental health in childhood

Table 3 displays the CR for psychological symptom scores in second-generation Irish children, relative to non-Irish children, in both cohorts. After adjusting for gender, second-generation Irish children had a higher psychological symptom count at ages 5 and 11 in the NCDS, and at age 16 in both cohorts. No interactions with gender were noted.

**Table 3:** Psychological health in second-generation Irish children, relative to the rest of cohort

*Age*	*Year*	**n**	*CR^a^*	*95% CI*	**P*-value*
NCDS (1958 British birth cohort)					
Age 7	1965	12 486	1.11	1.02 –1.20	0.01
Age 11	1969	12 244	1.09	1.00 –1.19	0.05
Age 16	1974	9906	1.23	1.10 –1.39	<0.001
BCS70 (1970 British birth cohort)					
Age 5	1975	11 168	1.02	0.96 –1.07	0.57
Age 10	1980	10 980	1.04	0.99 –1.09	0.16
Age 16	1986	6769	1.15	1.01 –1.31	0.03

^a^CR: psychological symptom count in Irish children relative to the rest of cohort. Models have been adjusted for gender.

### Does childhood material adversity or parental morbidity account for excess psychological morbidity in second-generation Irish children?

Table 4 displays the differences in psychological symptom counts in Irish compared with non-Irish children, after adjusting for covariates, in the NCDS. Adjusting for material hardship reduced the excess psychological symptom CR in Irish children, at ages 7 and 16.

**Table 4:** Psychological symptoms in second-generation Irish children relative to the rest of cohort; 1958 and 1970 British birth cohorts

	*1958 Birth cohort*								
	*Model 1*			*Model 2*			*Model 3*		
	*CR^a^*	*95% CI*	**P*-value*	*CR^a^*	*95% CI*	**P*-value*	*CR^a^*	*95% CI*	**P*-value*
Age 7 (1965); *N* = 9949									
Migration history									
Non-Irish	1.00	Ref.		1.00	Ref.				
Second-generation Irish	1.10	1.00 –1.21	0.04	1.05	0.96 –1.15	0.29			
Gender									
Male	1.00	Ref.		1.00	Ref.				
Female	0.72	0.69 –0.75	<0.001	0.71	0.69 –0.74	<0.001			
Mother's age									
Years	0.99	0.99 –1.00	<0.001 (trend)	1.00	0.99 –1.00	0.01 (trend)			
Mother's education									
Left school after 15	1.00	Ref.		1.00	Ref.				
Left school before 15	1.16	1.10 –1.22	<0.001	1.12	1.06 –1.18	<0.001			
Father's social class									
Non-manual	1.00	Ref.		1.00	Ref.				
Manual	1.22	1.16 –1.28	<0.001	0.86	0.82 –0.90	<0.001			
Contemporaneous hardship									
Hardship, 7^b^				1.13	1.10 –1.15	<0.001 (trend)			
Age 16 (1974); *N* = 7363									
Migration history									
Non-Irish	1.00	Ref.		1.00	Ref.		1.00	Ref.	
Second-generation Irish	1.22	1.06 –1.40	0.01	1.07	0.93 –1.22	0.36	1.19	1.03 –1.36	0.01
Gender									
Male	1.00	Ref.		1.00	Ref.		1.00	Ref.	
Female	0.80	0.75 –0.85	<0.001	0.80	0.75 –0.85	<0.001	0.80	0.76 –0.86	<0.001
Mother's age									
Years	0.99	0.99 –1.00	<0.001 (trend)	0.99	0.99 –1.00	0.04 (trend)	0.99	0.98 –0.99	<0.001 (trend)
Mother's education									
Left school after 15	1.00	Ref.		1.00	Ref.		1.00	Ref.	
Left school before 15	1.44	1.33 –1.55	<0.001	1.33	1.23 –1.44	<0.001	1.42	1.31 –1.53	<0.001
Father's social class									
Non-manual	1.00	Ref.		1.00	Ref.		1.00	Ref.	
Manual	1.46	1.35 –1.57	<0.001	1.35	1.25 –1.45	<0.001	1.44	1.34 –1.55	<0.001
Contemporaneous hardship									
Hardship, 16^b^				1.27	1.23 –1.31	<0.001 (trend)			
Parental chronic illness									
None							1.00	Ref.	
Illness							1.33	1.24 –1.44	<0.001
BCS70									
Age 16 (1986); *N* = 2464									
Migration history									
Non-Irish	1.00	Ref.		1.00	Ref.		1.00	Ref.	
Second-generation Irish	0.87	0.68 –1.10	0.24	0.85	0.67 –1.09	0.20	0.78	0.62 –1.00	0.05
Gender									
Male	1.00	Ref.		1.00	Ref.		1.00	Ref.	
Female	0.99	0.91 –1.06	0.73	0.99	0.91 –1.06	0.71	0.97	0.90 –1.05	0.47
Mother's age									
Years	0.99	0.99 –1.00	0.02 (trend)	0.99	0.98 –1.00	0.02 (trend)	0.99	0.98 –1.00	<0.001 (trend)
Mother's education									
Left school after 15	1.00	Ref.		1.00	Ref.		1.00	Ref.	
Left school before 15	1.06	0.99–1.15	0.10	1.04	0.96–1.12	0.34	1.02	0.95–1.10	0.52
Father' social class									
Non-manual	1.00	Ref.		1.00	Ref.		1.00	Ref.	
Manual	1.22	1.13 –1.32	<0.001	1.17	1.08 –1.26	<0.001	1.20	1.11 –1.29	<0.001
Contemporaneous hardship									
Hardship, 7^b^				1.10	1.06–1.14	<0.001 (trend)			
Maternal mental health									
Mother not distressed							1.00	Ref.	
Psychologically distressed							1.64	1.49–1.80	<0.001

Model 1: adjusted for gender, covariates from birth (mother's age, education, father's social class); Model 2: adjusted for gender, covariates from birth (mother's age, education, father's social class) and contemporaneous material hardship; Model 3: adjusted for gender, covariates from birth (mother's age, education, father's social class) and parental chronic health or maternal mental health.

^a^CR of psychological symptoms.

^b^Per SD increase in composite hardship variable.

At age 11 in the NCDS, accounting for variables from birth, contemporaneous material hardship or poorer parental health did not change the coefficient for association greatly (results available from author on request).

In the BCS70, after adjusting for maternal psychological distress, covariates from birth and material hardship, second-generation Irish children had a psychological symptom count 0.78 times less than non-Irish children (95% CI: 0.62–1.00; Table 4).

## Discussion

### Main findings

We analysed data from two nationally representative British birth cohorts, covering the first 16 years of children's lives. In both cohorts, second-generation Irish children grew up under conditions of marked material adversity, compared with children without a parental history of migration. In the NCDS, Irish children had poorer psychological health at ages 7, 11 and 16 compared with that in the rest of the cohort. In the BCS70, Irish children had both emotional and behavioural problems at age 16.

In the NCDS, exposure to material adversity accounted for much of the excess psychological morbidity in Irish children, relative to other cohort members. In the BCS70, Irish-born mothers were also more likely to screen positive for high levels of psychological distress, when their child was aged 5 or 10. Adjusting for maternal mental health led to a finding of slightly better mental health in second-generation Irish 16 year olds, compared with the rest of the sample, in the BCS70. It is noteworthy that the greater risk of mental and physical health problems in Irish-born parents was also partially or fully accounted for by material adversity indicators. The findings from the current analysis highlight the importance of material adversity in the post-migration settlement context as also being important for observed health inequalities in Irish-born people. Given the consistency of associations observed across cohort members' early years, material adversity may be a common factor accounting for the health disadvantages of second-generation Irish children and in their parents.

### What is already known and what this study adds

The findings implicate important risk factors for migration and childhood mental health, observed in two nationally representative birth cohorts separated by more than a decade. A recent systematic review indicated a complex picture of heterogeneity in the contemporary prevalence of mental health conditions in ethnic minority children growing up in Britain, with some groups experiencing better mental health and some groups experiencing a more disadvantaged mental health profile, relative to white British children.^1^ Very few studies had directly examined parental health or material hardship as putative mediating factors.^1^ Although the context of Irish migration to Britain has changed over the last 50 years, future research could assess if these risk factors are still relevant to second-generation Irish children growing up in Britain today, or if these factors have salience for the mental health of other second-generation ethnic minority children living in Britain.

Previous studies have shown that Irish-born adults living in Britain have an elevated prevalence of mental health problems,^9,10^ associated with unplanned migration and poorer social support.^10^ The findings from the present study indicate that second-generation Irish children also experienced an elevated prevalence of mental health problems; however, these mental health problems were mediated by the poorer psychological health of their mothers. Of note, material hardship mediated mental health problems in both Irish children and in their mothers, as well as chronic health problems in Irish-born parents. This suggests possible points for intervention, in the post-migration settlement context, which may be of benefit to migrant families.

The finding at age 16 that mental health in Irish children was better than the rest of the BCS70, after adjusting for maternal mental health and material hardship, has also been noted for other ethnic minority groups.^1^ The main difference in the present study and previous work was that this was observed only ‘after’ taking into account for maternal mental health and material hardship, and not ‘in spite of’ living in socially disadvantaged circumstances.^1^

Childhood mental health may act as a distal risk factor for adult common mental disorders.^3^ As it is known that Irish people living in Britain suffer an elevated prevalence of common mental disorders in adulthood,^9,25^ the findings from the present study hint at potential life-course mechanisms for the ‘intergenerational transmission’ of poorer mental health in this group.^25^

### Strengths and limitations

A strength of the dataset derives from its prospective design, with detailed, regular assessment of contemporaneous social circumstances experienced by cohort members as they grew up, together with the use of validated instruments to assess psychological health. Most measures were prospective and, therefore, less prone to measurement error. An exception was for the ‘parental migration history’ question, which retrospectively asked after the country of birth of parents. It is reassuring that kappa assessing the reliability of responses to this question, over two consecutive sweeps, was high.

In the NCDS, the question on parental origins was asked when children were aged 11 or 16. We could not ascertain the parental origins of cohort members already lost to follow-up by this point. Conversely, although the question on parental origins was asked at birth in the BCS70, a slightly larger proportion of Irish respondents refused participation at the later sweeps of the survey. We cannot know how this may have biased overall findings, although it is likely that this would have weakened effects. A further concern relates to the age 16 sweep of the BCS70, where a large proportion of data were missing due to the teacher's strike, that year.^24^ This loss of information may have impacted on the power to detect differences in the sample—however, despite this, second-generation Irish children continued to show social and mental health inequalities compared with the rest of the cohort at this age. The use of two cohorts, suggesting relatively consistent results across time, lends support to the notion that mechanisms underlying missing data have not overtly impacted on overall conclusions.

Measures for parental health prior to migration were not available, and it was not possible to assess whether mental health problems in the parents were a consequence of adversity related to settlement,^25,26^ or alternatively, factors pre-dating migration.^10^ A social rating of neighbourhoods was taken at age 5 in the BCS70. Although assessed by an independent observer, as this was a subjective measure, this would have been a weaker assessment of area-level disadvantage, compared with measures commonly in use today such as the Index of Multiple Deprivation.^27^

A further limitation of the present study related to the period effects from using data with its origins in the late 1950s and early 1970s. As the cohorts were nationally representative of children born in Britain, a degree of generalizability to other children born at this time could be inferred, and the findings may implicate important risk factors for migration and childhood mental health. However, the circumstances surrounding migration and settlement of Irish families to Britain have been changed markedly over the last 50 years, and so one should use a degree of caution in extrapolating the findings outside of this context.

## Supplementary data

Supplementary data are available at *Journal of Public Health* online.

## Funding

This work was supported by the Medical Research Council (MRC), in the form of a fellowship awarded to J.D.-M. (grant no. G0701595/1). The funder did not play any part in design of the research protocol, data analysis or preparation of the report. The analyses in this work are based wholly or in part on analysis of data from the NCDS and the BCS70. The data were deposited at the UK Data Archive by the Centre for Longitudinal Studies at the Institute of Education, University of London. NCDS is funded by the Economic and Social Research Council.

## Supplementary Material

Supplementary Data
