# Supplementary material for: Born into adversity: psychological distress in two birth cohorts of second-generation Irish children growing up in Britain
Source: J Public Health (Oxf). 2013 Apr 17;36(1):92–103. doi: 10.1093/pubmed/fdt034 (PMC3935491; doi:10.1093/pubmed/fdt034)
Supplement: Supplementary Data [file supp_36_1_92__index.html]

Born into adversity: psychological distress in two birth cohorts of second-generation Irish children growing up in Britain — Born into adversity: psychological distress in two birth cohorts of second-generation Irish children growing up in Britain — Supplementary Data 

# Born into adversity: psychological distress in two birth cohorts of second-generation Irish children growing up in Britain

## Supplementary Data

Supplementary Data

**Files in this Data Supplement:**

- Supplementary Data - Docx file
