# Supplementary material for: Born into adversity: psychological distress in two birth cohorts of second-generation Irish children growing up in Britain
Source: J Public Health (Oxf). 2013 Apr 17;36(1):92–103. doi: 10.1093/pubmed/fdt034 (PMC3935491; doi:10.1093/pubmed/fdt034)
Supplement: Supplementary Data [file supp_fdt034_fdt034supp.docx]

**APPENDIX; ONLINE REPOSITORY MATERIAL**

**Table 1:** Reasons for non-response across sweeps

|  | **Country of birth (COB) of cohort members' parents** | | | | | |
| --- | --- | --- | --- | --- | --- | --- |
|  | **Both parents born in England, Scotland or Wales** | | **One or both parents born in Ireland/ N. Ireland** | | **No information on COB of cohort member’s parent^a^** | |
| **NCDS**  **N (%)** | **12933** | **(%)** | **791** | **(%)** | **3041** | **(%)** |
| **Sweep 0 (birth)** |  |  |  |  |  |  |
| some data | 12769 | 99% | 782 | 99% | 3002 | 99% |
| no data/ other contact later | 164 | 1% | 9 | 1% | 39 | 1% |
| **Sweep 1 (Age 7)** |  |  |  |  |  |  |
| some data | 12144 | 94% | 710 | 90% | 1404 | 46% |
| no data dead | 0 | 0% | 0 | 0% | 812 | 27% |
| no data emigrant | 94 | 1% | 10 | 1% | 353 | 12% |
| No data- refusal/ other | 695 | 5% | 71 | 9% | 472 | 16% |
| **Sweep 2 (Age 11)***^a^* |  |  |  |  |  |  |
| some data | 12447 | 96% | 761 | 96% | 707 | 23% |
| no data dead | 0 | 0% | 0 | 0% | 829 | 27% |
| no data emigrant | 68 | 1% | 6 | 1% | 586 | 19% |
| No data- refusal/ other | 418 | 3% | 24 | 3% | 919 | 30% |
| **Sweep 3 (Age 16)***^a^* |  |  |  |  |  |  |
| some data | 11729 | 91% | 699 | 88% | 710 | 23% |
| no data dead | 24 | 0% | 1 | 0% | 835 | 27% |
| no data emigrant | 90 | 1% | 18 | 2% | 603 | 20% |
| No data- refusal/ other | 1090 | 8% | 73 | 9% | 893 | 29% |
| **BCS70** |  |  |  |  |  |  |
| **N (%)** | **14022** | **(%)** | **832** | **(%)** | **737** | **(%)** |
| **Birth** *^a^* |  |  |  |  |  |  |
| some data | 14022 | 100% | 832 | 100% | 21 | 3% |
| no data- refusal/ other | 0 | 0% | 0 | 0% | 716 | 97% |
| **Sweep 1 (Age 5)** |  |  |  |  |  |  |
| some data | 11147 | 79% | 544 | 65% | 257 | 35% |
| no data-dead | 474 | 3% | 31 | 4% | 3 | 0% |
| no data- refusal/ other | 2401 | 17% | 257 | 31% | 477 | 65% |
| **Sweep 2 (Age 10)** |  |  |  |  |  |  |
| some data | 12013 | 86% | 563 | 68% | 685 | 93% |
| no data-dead | 490 | 3% | 31 | 4% | 3 | 0% |
| no data- refusal/ other | 1519 | 11% | 238 | 29% | 49 | 7% |
| **Sweep 3 (Age 16)** |  |  |  |  |  |  |
| some data | 9502 | 68% | 388 | 47% | 540 | 73% |
| no data-dead | 498 | 4% | 31 | 4% | 4 | 1% |
| no data- refusal/ other | 4022 | 29% | 413 | 50% | 193 | 26% |

***Key:*** *^a^ This information was missing either because the cohort member was not present when this question was asked (sweeps 2 and 3 in NCDS and at birth in BCS70), or because parents were present but a response was not encoded.*

**APPENDIX; ONLINE REPOSITORY MATERIAL**

**Table 2:** Characteristics of cohort members missing information on parental

Country of birth in NCDS

|  | **Information on parental COB available** | | **No information on parental COB available** | |
| --- | --- | --- | --- | --- |
|  | **N** | **(%)** | **N** | **(%)** |
| **Gender** |  |  |  |  |
| male | 7385 | 51% | 836 | 52% |
| female | 7059 | 49% | 767 | 48% |
| **Social class at birth** |  |  |  |  |
| I-II | 2346 | 16% | 291 | 18% |
| III | 8197 | 57% | 828 | 52% |
| IV-V | 3014 | 21% | 314 | 20% |
| sick/dead/retired/student/no male head/ no answer | 703 | 5% | 131 | 8% |
| *missing* | *184* | 1% | *39* | 2% |
| **Housing tenure (age 7)** |  |  |  |  |
| owner occupied | 5409 | 37% | 388 | 24% |
| council rented | 5262 | 36% | 341 | 21% |
| private rented | 1554 | 11% | 141 | 9% |
| rent free | 298 | 2% | 14 | 1% |
| other | 993 | 7% | 231 | 14% |
| *missing* | *928* | 6% | *488* | 30% |
| **Tenure, age 16** |  |  |  |  |
| owner occupied | 5494 | 38% | 3 | 0% |
| council | 4612 | 32% | 9 | 1% |
| private rented | 561 | 4% | 0 | 0% |
| tied | 414 | 3% | 0 | 0% |
| other | 41 | 0% | 1 | 0% |
| *missing* | *3322* | 23% | *1590* | 99% |

*Excludes children known to have died or emigrated by age 16*

**APPENDIX; ONLINE REPOSITORY MATERIAL**

**Table 3:** Characteristics of cohort members missing information on parental

country of birth in BCS70

|  | **Information on parental COB** | | **No information on parental COB** | |
| --- | --- | --- | --- | --- |
|  | **N** | **(%)** | **N** | **(%)** |
| **Gender** |  |  |  |  |
| male | 7733 | 52% | 378 | 51% |
| female | 7115 | 48% | 357 | 48% |
| *missing* | *6* | *0%* | *2* | *0%* |
| **Social class at birth** | | | | |
| I-II | 2326 | 16% | 1 | 0% |
| IIINM | 1717 | 12% | 2 | 0% |
| IIIM | 6758 | 45% | 6 | 1% |
| IV | 1996 | 13% | 1 | 0% |
| V | 913 | 6% | 1 | 0% |
| other/ unsupported | 1070 | 7% | 6 | 1% |
| *missing* | *74* | *0%* | *720* | *98%* |
| **Tenure at age 5 (1975)** | | | | |
| Owner/occupier/being bought | 6531 | 44% | 142 | 19% |
| private rented | 691 | 5% | 20 | 3% |
| council | 3913 | 26% | 74 | 10% |
| other | 566 | 4% | 19 | 3% |
| *missing* | *3153* | *21%* | *482* | *65%* |
| **Tenure at 16 (1986)** |  |  |  |  |
| Owner occupier | 3578 | 24% | 178 | 24% |
| Private rented | 131 | 1% | 4 | 1% |
| Council | 738 | 5% | 48 | 7% |
| Other | 86 | 1% | 4 | 1% |
| *missing* | *10321* | *69%* | *503* | *69%* |

*Excludes children known to have died or emigrated by age 16*

| **APPENDIX; ONLINE REPOSITORY MATERIAL**  **Table 4:** Mean scores (with standard deviations) on composite hardship variables by parental migration status. Composite hardship variables were derived through principal components analysis | | | | | | | |
| --- | --- | --- | --- | --- | --- | --- | --- |
| ***1958 British Birth Cohort*** | | | | | | | |
|  |  | **Mean** | **SD** | ***Variables comprising composite hardship variable*** | | |  |
| *Age 7* |  |  |  |  |  |  |  |
| Parents' country of birth | England, Scotland, Wales | -0.04 | 1.24 | *Lack of access to one or more household amenities ^a^, household overcrowding, family financial difficulties, family housing difficulties* | | |  |
|  | Irish-born | 0.58 | 1.66 |  |  |  |  |
| *Age 11* |  |  |  |  |  |  |  |
| Parents' country of birth | England, Scotland, Wales | -0.04 | 1.25 | *Lack of access to one or more household amenities ^a^, household overcrowding, serious financial hardship in the last year, free school meals* | | |  |
|  | Irish-born | 0.37 | 1.43 |  |  |  |  |
| *Age 16* |  |  |  |  |  |  |  |
| Parents' country of birth | England, Scotland, Wales | -0.05 | 1.23 | *Lack of access to one or more household amenities ^a^, household overcrowding, serious financial hardship in the last year, free school meals* | | |  |
|  | Irish-born | 0.47 | 1.52 |  |  |  |  |
| ***1970 British Birth Cohort*** | | | | | | | |
|  |  | **Mean** | **SD** | ***Variables comprising composite hardship variable*** | | |  |
| *Age 5* |  |  |  |  |  |  |  |
| Parents' country of birth | England, Scotland, Wales | -0.06 | 1.2 | *Lack of access to one or more household amenities ^a^, household overcrowding, car/ van ownership, social rating of neighbourhood (well to do vs poor/ average/ rural)* | | |  |
|  | Irish-born | 0.42 | 1.23 |  |  |  |  |
|  |  |  |  |  |  |  |  |
| *Age 10* |  |  |  |  |  |  |  |
| Parents' country of birth | England, Scotland, Wales | -0.06 | 1.32 | *Car/ van ownership, damp in housing, free school meals, resident in council housing* | | |  |
|  | Irish-born | 0.61 | 1.52 |  |  |  |  |
|  |  |  |  |  |  |  |  |
| *Age 16* |  |  |  |  |  |  |  |
| Parents' country of birth | England, Scotland, Wales | -0.01 | 1.3 | *Car ownership, damp in housing, resident in council housing, unable to afford to heat whole house in winter* | | |  |
|  | Irish-born | 0.18 | 1.46 |  |  |  |  |
| ***Key*** |  |  |  |  |  |  |  |
| *^a^ Lack of access to any one of indoor bathroom, hot water or indoor toilet* | | | | | | | |
